# Does the Presence of Psychiatric Symptoms in Adolescents With Special Educational Needs at Certain Time Points in Earlier Life Predict Functional Outcome Later On?

**DOI:** 10.1192/bjo.2022.174

**Published:** 2022-06-20

**Authors:** Neha Bansal, Lindsay Mizen, Andrew Stanfield

**Affiliations:** 1 NHS Lothian, Edinburgh, United Kingdom; 2 University of Edinburgh, Edinburgh, United Kingdom

## Abstract

**Aims:**

This study analyses the progression of psychiatric symptoms over time of young people with special educational needs (SEN). The aims of this study were: 1) To examine whether the presence of psychiatric symptoms in earlier life are more likely to impact functional outcomes in later life in those with SEN; 2) Whether the presence of psychiatric symptoms in adolescence predicts functional outcomes in early adult life.

**Methods:**

Data were obtained from the Edinburgh Study of Comorbidity (ESC) which was a longitudinal follow-up study of adolescents with SEN. This study had ethical approval from the Multicentre Research Ethics Committee for Scotland. It involved head teachers of 99 schools around Scotland identifying pupils aged 13–22 years whom they would estimate as functioning in the borderline to mild intellectual disability range (estimated IQ between 50–80) and were therefore receiving special educational assistance.

Adolescents with SEN were assessed with the Clinical Interview Schedule (CIS) to evaluate the presence of psychiatric symptoms. A total of 247 individuals with SEN were recruited to the study. They completed the CIS at baseline (T1), 1–2 years later (T2) and 6 years later (T3). At T3, the participants also completed the World Health Organisation Disability Assessment Schedule 2.0 (WHO-DAS) to measure the degree of functional impairment. Correlation statistical analyses were carried out to find whether there was a significant relationship between CIS and total WHO-DAS scores.

**Results:**

There was a statistically significant correlation between total WHO-DAS score with slowness and anxiety symptoms (p values 0.008 and 0.024 respectively) measured on the CIS at T1. None of the symptoms measured on CIS at T2 had a statistically significant correlation with total WHO-DAS score. With the symptoms that were significant, after application of a Bonferroni correction, none of the symptoms measured on CIS had a statistically significant correlation at any time point with total WHO-DAS score.

**Conclusion:**

Our results show that there is some evidence that anxiety and slowness in adolescence are associated with greater functional impairment in young adulthood. However, further research is required to confirm this relationship. Our data highlight the potential value of identification and treatment of psychiatric symptoms in early adolescence.

